# Curcumin Analog DK1 Induces Apoptosis in Human Osteosarcoma Cells In Vitro through Mitochondria-Dependent Signaling Pathway

**DOI:** 10.3390/molecules23010075

**Published:** 2018-01-05

**Authors:** Muhammad Nazirul Mubin Aziz, Yazmin Hussin, Nurul Fattin Che Rahim, Noraini Nordin, Nurul Elyani Mohamad, Swee Keong Yeap, Chean Yeah Yong, Mas Jaffri Masarudin, Yoke Kqueen Cheah, Nadiah Abu, Muhammad Nadeem Akhtar, Noorjahan Banu Alitheen

**Affiliations:** 1 Department of Cell and Molecular Biology, Faculty of Biotechnology and Biomolecular Sciences, Universiti Putra Malaysia, UPM, Serdang 43400, Selangor, Malaysia; muhammadnazirulmubin@gmail.com (M.N.M.A.); yazminh93@gmail.com (Y.H.); nurulfattincherahim@gmail.com (N.F.C.R.); noraininordin1303@gmail.com (N.N.); elyani.mohamad@gmail.com (N.E.M.); masjaffri@upm.edu.my (M.J.M.); 2 China-ASEAN College of Marine Sciences, Xiamen University Malaysia, Sepang 43900, Selangor, Malaysia; skyeap2005@gmail.com; 3 Department of Microbiology, Faculty of Biotechnology and Biomolecular Sciences, Universiti Putra Malaysia, UPM, Serdang 43400, Selangor, Malaysia; yongcheanyeah@hotmail.com; 4 Department of Biomedical Science, Faculty of Medicine and Health Science, Universiti Putra Malaysia, UPM, Serdang 43400, Selangor, Malaysia; ykcheah@upm.edu.my; 5 UKM Medical Molecular Biology Institute (UMBI), UKM Medical Centre, Cheras, Kuala Lumpur 56000, Malaysia; nadyaboo@gmail.com; 6 Faculty of Industrial Sciences & Technology, Universiti Malaysia Pahang, Lebuhraya Tun Razak 26300, Kuantan Pahang, Malaysia; nadeemupm@gmail.com

**Keywords:** curcumin analog DK1, human osteosarcoma U-2OS, MG-63

## Abstract

Osteosarcoma is one of the primary malignant bone tumors that confer low survival rates for patients even with intensive regime treatments. Therefore, discovery of novel anti-osteosarcoma drugs derived from natural products that are not harmful to the normal cells remains crucial. Curcumin is one of the natural substances that have been extensively studied due to its anti-cancer properties and is pharmacologically safe considering its ubiquitous consumption for centuries. However, curcumin suffers from a poor circulating bioavailability, which has led to the development of a chemically synthesized curcuminoid analog, namely (*Z*)-3-hydroxy-1-(2-hydroxyphenyl)-3-phenylprop-2-en-1-one (DK1). In this study, the cytotoxic effects of the curcumin analog DK1 was investigated in both U-2OS and MG-63 osteosarcoma cell lines using 3-(4,5-dimethylthiazol-2-yl)-2,5-diphenyltetrazolium bromide (MTT) assay and cell death was microscopically examined via acridine orange/propidium iodide (AO/PI) double staining. Flow cytometer analysis including Annexin V/Fluorescein isothiocyanate (FITC), cell cycle analysis and JC-1 were adapted to determine the mode of cell death. Subsequently in order to determine the mechanism of cell death, quantitative polymerase chain reaction (qPCR) and proteome profiling was carried out to measure the expression of several apoptotic-related genes and proteins. Results indicated that DK1 induced U-2 OS and MG-63 morphological changes and substantially reduced cell numbers through induction of apoptosis. Several apoptotic genes and proteins were steadily expressed after treatment with DK1; including caspase 3, caspase 9, and BAX, which indicated that apoptosis occurred through a mitochondria-dependent signaling pathway. In conclusion, DK1 could be considered as a potential candidate for an anti-osteosarcoma drug in the near future, contingent upon its ability to induce apoptosis in osteosarcoma cell lines.

## 1. Introduction

Osteosarcoma (OS) is one of the primary malignant bone tumors that is usually diagnosed among children and adolescents [1]. In Malaysia alone, 3% from a total of 800 children were diagnosed with OS, which frequently occurs among boys. Even with an intensive regime treatment that incorporates surgical and chemotherapy, the survival rate for OS significantly drops to 30% in the patients diagnosed with metastatic OS [1,2]. Although current chemotherapy drugs are potent enough to increase a patients’ survival rate, their current administration suffers from several side effects. For example, Doxorubicin is a chemotherapy drug usually administered to OS patients. However, it poses major side effects including mucositis, myelosuppression, and systemic toxicity such as cardiotoxicity at high dosage of utilization [3,4]. With these inherent side effects, younger patients such as children diagnosed with OS are more susceptible to efficacy during treatment thus reducingtheir survival rate tremendously [5]. Cancer is caused by the irregular activity of the cell cycle and has the capability to disrupt the apoptosis signaling pathways [6,7]. Therefore, discovery of novel drugs derived from natural products like curcumin that are able to target cell cycle progression and induce apoptosis specifically on osteosarcoma without causing non-specific efficacy to normal cells, is highly desired [8].

**Figure 1:**
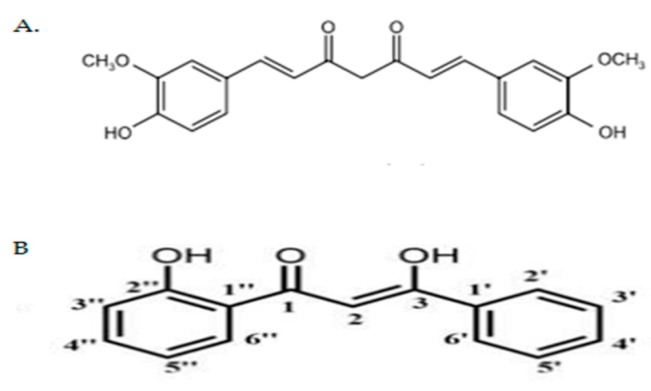
(**A**) Chemical structure of natural curcumin [11]; (**B**) Chemical structure of curcumin analog DK1 [6].

Curcumin (Figure 1A) is an active component isolated from turmeric, a spice originating from the roots of *Curcuma longa* and has been used for centuries in most Asian countries [9]. This natural substance is widely known for its broad spectrum of biological activities, including anti-cancer, anti-oxidant, anti-inflammatory, anti-angiogenic, and anti-proliferative properties [9,10]. Considering that it is pharmacologically safe for consumption and possesses anti-cancer activities, curcumin potentially is a good candidate for the development of an innovative anti-osteosarcoma drug [8,10]. Therefore, in the past decade, many synthetic compounds derived from curcumin were synthesized. These curcumin analogs and derivatives have been shown to improve certain physiological properties, such as cytotoxic, and anti-inflammatory effects as well as anti-tumoral activities that in turn increased curcumin’s potential as a therapeutic agent for anti-cancer treatment [8].

In this study, a curcumin analog namely (*Z*)-3-hydroxy-1-(2-hydroxyphenyl)-3-phenylprop-2-en-1-one (DK1) (Figure 1B) was synthesized to improve the bioavailability of dietary curcumin that has limited efficacy due to its poor bioavailability [6,7,8]. Additionally, DK1 also has been reported to show cytotoxic activity in MCF-7 breast cancer cell line by inducing G2/M cell cycle arrest and also induced apoptosis [6]. DK1 was synthesized in the form of 100% pure crystal by using the Baker–Venkataraman Rearrangement method and was further confirmed via single X-ray analysis [6]. This study was carried out primarily to test whether the synthetically synthesized curcumin analog DK1 can broaden its anti-cancer activity to other cancer types like osteosarcoma, by selectively inducing apoptosis on the osteosarcoma cell lines without affecting the function of the normal cells.

## 2. Results

### 2.1. DK 1 Inhibits the Proliferation of U-2OS and MG-63

MTT assay was conducted to assess the anti-proliferative effects of curcumin analog DK1 on osteosarcoma cell lines and normal fibroblast cell line 3T3 [12]. The cells were treated with 2-fold serial dilution of DK1 for 48 h. Table 1 represents the cytotoxic effects exhibited by DK1 48 h post treatment at 50% inhibition concentrations (IC_50_). Based on Table 1 the effects of curcumin analog DK1 were found to confer a dose dependent manner with 50% of cell viability and were inhibited at concentrations lower than 30 µM. The IC_50_ value of DK1 is lower in U-2OS (19.6 µM) compared to MG-63 (23.8 µM), however there was no IC_50_ obtained for 3T3 normal cell line; which indicated that DK1 did not interfere with the proliferation of normal fibroblast cell lines.

### 2.2. DK1 Altered the Morphological Appearances and Induces Apoptosis in U-2OS and MG-63

**Figure 2:**
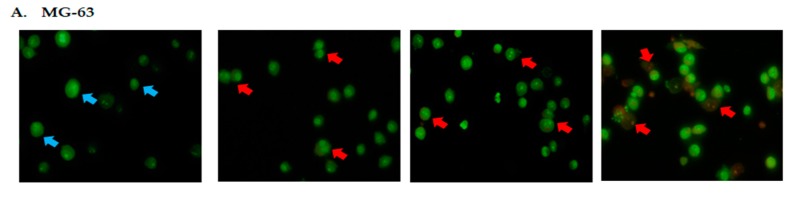

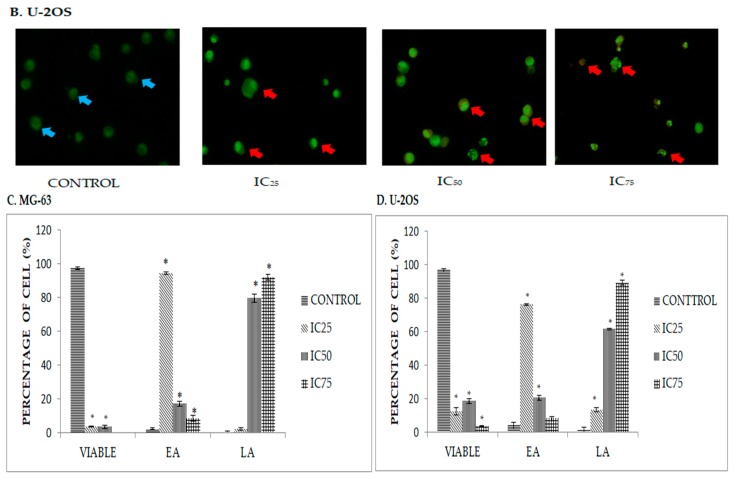
(**A**) Distinct morphological changes in MG-63 after 48 h of treatment with DK1 (IC_25_ (6.6 µM), IC_50_ (23.8 µM), and IC_75_ (30 µM)); (**B**) Distinct morphological changes in U-2OS after 48 h treatment with DK1 (IC_25_ (2.2 µM), IC_50_ (19.6 µM) and IC_75_ (30 µM)); (**C**,**D**) Quantification analysis of MG-63 and U-2OS based on cells uptake of acridine orange and propidium iodide in 200 cells. (Blue arrow: viable; red arrow: apoptosis). EA (early apoptosis), LA (late apoptosis). All data are expressed as mean ± standard error mean (S.E.M). * *p* < 0.05 compared with corresponding controls (Magnification: 200×).

Acridine orange/propidium iodide (AO/PI) double staining assay was conducted to examine the cell death of osteosarcoma cells microscopically. By using this staining assay, the morphology and cellular profiles for viable, apoptotic and necrotic cells can be distinguished [13]. Viable cells with intact membranes will stain green, whilst early apoptotic cells will stain with a green color but are distinguished by occurrences of distinct features such as membrane disruption, while late apoptosis/necrosis will stain red [13]. Around 200 populations of cells were quantified for statistical analysis [13]. Based on MTT assay, three different inhibition concentrations (IC_25_, IC_50_, and IC_75_) for DK1 were obtained and subsequently used to treat both MG-63 and U-2OS cell lines for 48 h. Figure 2 represents the fluorescence photomicrographs of MG-63 and U-2OS osteosarcoma cell lines 48 h post treatment with three different concentrations of DK1. Based on Figure 2 around 90% of viable cells were prominent in control samples of both MG-63 and U-2OS. However, when both cell lines were exposed to DK1, the viable cells’ percentage significantly decreased below 20% viability. At 48 h post-treatment for the MG-63 and U-2OS cell lines, the increment of apoptotic cells was found to be in a dose-dependent manner. Based on Figure 2A,B early apoptotic features can be clearly observed even at the lowest concentration of IC_25_. These results may suggest that DK1 is capable of inducing apoptosis in MG-63 and U-2 OS at all concentrations and the percentage of apoptotic cells increases with dosages of IC_50_ and IC_75_.

### 2.3. Quatification of Apoptotic Cell Death upon Exposure to DK1 via Annexin V/FITC Binding Assay

**Figure 3:**
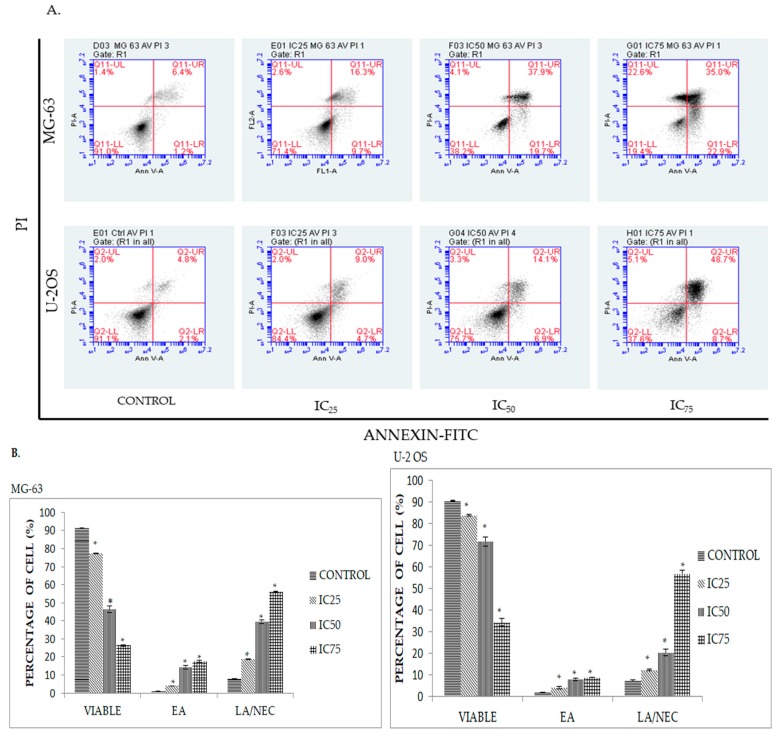
(**A**) Histogram analysis of Annexin V/ FITC in MG-63 and U-2OS after being treated with three different concentration of DK1 (IC_25_, IC_50_, IC_75_) for 48 h. There are four quadrants in the histogram with different quadrants indicating different types of cell population; LL (viable), LR (early apoptosis), UR (late apoptosis), UL (necrosis); (**B**) Quantification analysis of MG-63 and U-2OS based on percentage of cells that undergo apoptosis. EA (early apoptosis), LA (late apoptosis), NEC (necrosis). All data are expressed as mean ± standard error mean (S.E.M). * *p* < 0.05 compared with corresponding controls.

Induction of apoptosis is one of the key areas of interest in development of candidate drugs against cancer [14]. In order to quantify the apoptotic activity of cancer cells when exposed to DK1 treatment, Annexin V/FITC binding assay which detects the translocation of phosphatidylserine in cells was applied [15]. Commonly, phosphatidylserine is restricted to inside of viable cells. However, upon treatment with DK1 the membrane of the cell disintegrated and exposed the phosphatidylserine extracellularly [16]. Externalization of this phosphatidylserine can be detected by conjugation with Annexin V/FITC binding dye [16]. This reliable method can then be used to differentiate between viable cells (annexin V-FITC−/PI−), early apoptosis (annexin V-FITC+/PI−), and late apoptosis/necrosis (annexin V-FITC+/PI+). Figure 3 shows the representative plot of Annexin V-FITC assay 48 h post treatment with DK1 towards osteosarcoma cell lines. Based on Figure 3A, a pattern of cell population shiftting from viable to early apoptosis to late apoptosis/necrosis in both MG-63 and U-2OS was observed. The percentage of early apoptotic cell in MG-63 increased gradually from 0.8% in the control group to 16.5% in the IC_75_ treatment group. A similar pattern was also exhibited in U-2OS treated groups, where the percentage of early apoptotic cells gradually increased from 2.1% in the control group to 8.7% in the IC_75_ treatment group. A similar pattern was noticed in late apoptosis/necrosis cells as well. Based on the statictical analysis it can be concluded that there is a direct relationship that is proportional between the percentage of cell viability and the dosing of DK1.

### 2.4. DK1 Induces Cell Cycle Accumulation at S Phase in MG-63 and U-2OS

**Figure 4:**
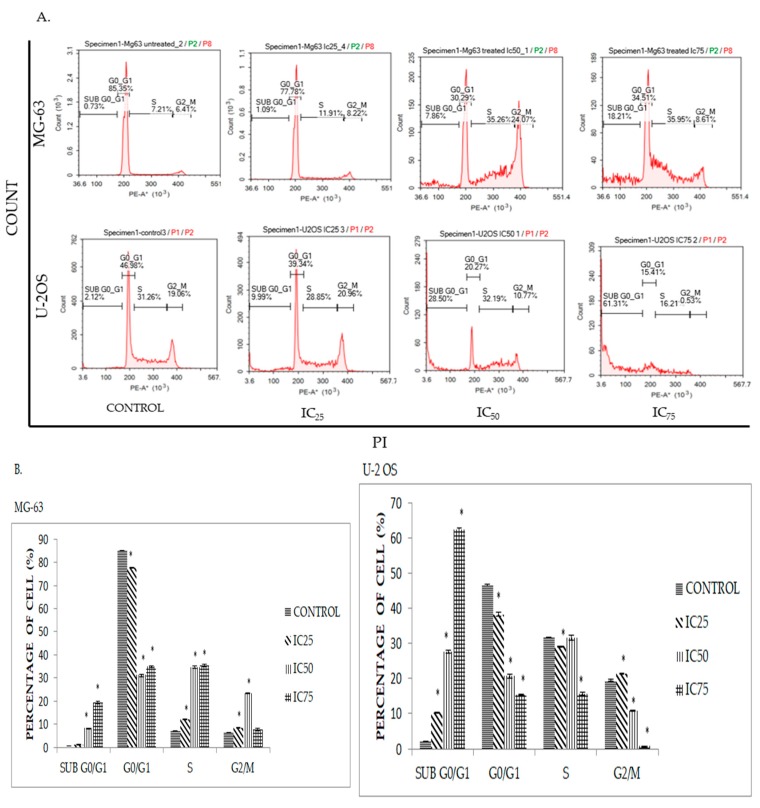
(**A**) Cell cycle histogram analysis for MG-63 and U-2OS after being treated with three different concentrations of DK1 (IC_25_, IC_50_, IC_75_) at 48 h; (**B**) Quantification analysis of cell cycle distribution for MG-63 and U-2OS when treated with three different concentrations of DK1. All data are expressed as mean ± standard error mean (S.E.M). * *p* < 0.05 compared with corresponding controls.

Cancer cells are known to undergo an irregular cell cycle progression due to mutations that occur in their genetic code and the abundance of growth factors surrounding it [6,17]. In order to disrupt this process, DK1 dysregulates cell cycle activity by interrupting the cell cycle checkpoint, rendering the cell more susceptible to damage [17]. In order to determine whether DK1 is able to interfere with cell cycle progression, cell cycle analysis was conducted through DNA staining with PI. Shown in Figure 4, the percentage of cells undergoing sub G0/G1 phase reflecting apoptotic cells in both cell lines MG-63 and U-2OS gradually increased to 18% and 61% respectively, as compared to the control when exposed to three different concentrations of DK1 (IC_25_, IC_50_, IC_75_) for 48 h. However, significant cell cycle arrest at S phase was only observed in MG-63 compared to U-2OS.

### 2.5. DK 1 Induces Changes in the Mitochondrial Membrane Potential of U-2OS and MG-63

**Figure 5:**
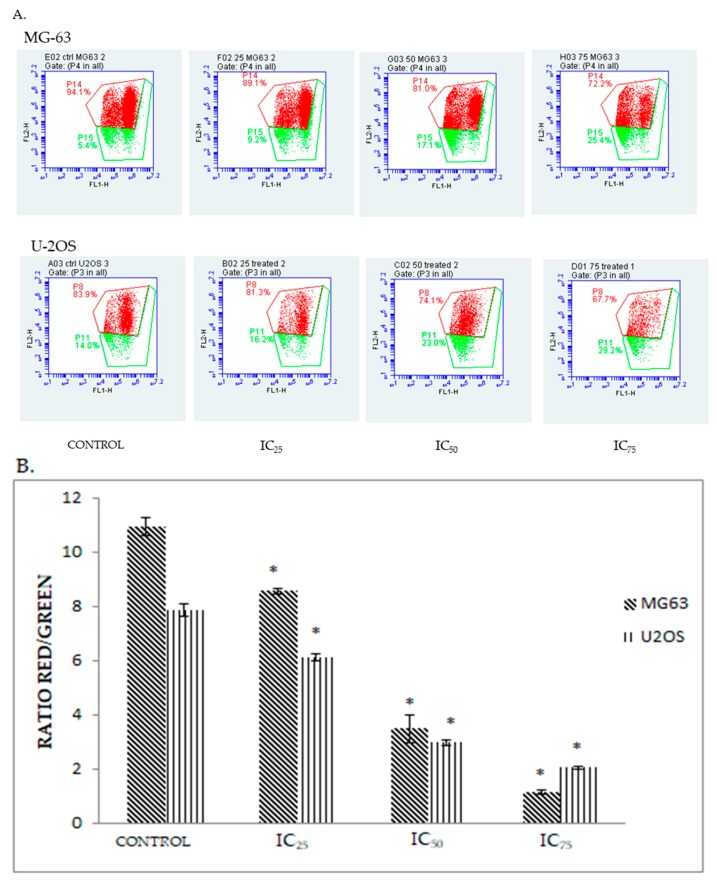
(**A**) Histogram analysis of the depolarization of mitochondrial membrane potential for MG-63 and U-2OS after being treated with three different concentrations of DK1 (IC_25_, IC_50_, IC_75_) at 48 h; (**B**) Quantification analysis of mitochondrial membrane analysis for MG-63 and U-2OS when treated with three different concentrations of DK1. All data are expressed as mean ± standard error mean (S.E.M). * *p* < 0.05 compared with corresponding controls.

Mitochondrial membrane permeabilization is one of the important key features of mitochondrial apoptotic pathway [18]. To measure the mitochondrial membrane potential, U-2OS and MG-63 cell lines were treated with DK1 and exposed to JC-1 dye; a compound that emits red fluorescence when present as aggregates, but fluoresces green when existing as monomers [19]. The ratio of red fluorescence to green florescence is therefore directly proportional to the strength of the mitochondrial membrane potential (ΔΨm) [19]. Healthy cells will show red fluorescence detected as aggregates whereas in the apoptotic cells JC-1 exists in monomeric form and they are detected as green fluorescence. In DK1-treated a shift between the percentage of aggregates and monomers was reflected upon increasing the dose from IC_25_ to IC_75_ [19]. The higher the dose of the DK1 treatment, the lower the ratio of aggregates to monomers observed as shown in Figure 5A. These results clearly showed that the induction of apoptosis by DK1 occurred in a dose dependent manner. Thus, this histogram analysis manifested that DK1 induced changes in the mitochondrial membrane potential in both U-2OS and MG-63.

### 2.6. DK1 Regulates Several Apoptosis and Cell Cycle Related Genes and Protein

**Figure 6:**
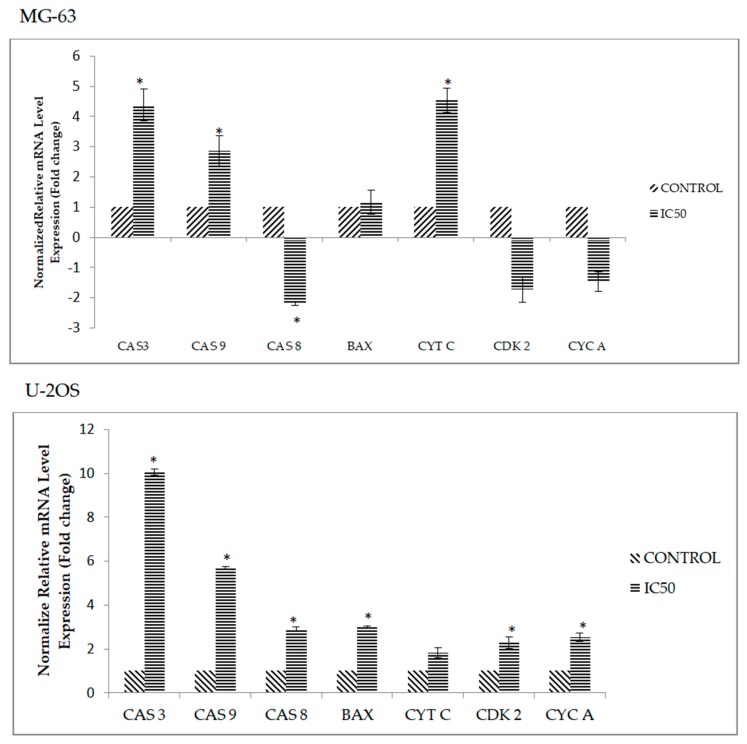
qPCR analysis of apoptosis and cell cycle related genes; caspase 3 (CAS 3), caspase 8 (CAS 8) and caspase 9 (CAS 9), BAX, cytochrome c (CYT C), Cdk 2 (CDK 2), cyclin A (CYC A) for MG-63 and U-2OS when treated DK1 (IC_50_) for 48 h. Expressions of all target genes were normalized to ACTB and 18srRNA. All data are expressed as mean ± standard error mean (S.E.M). * *p* < 0.05 compared with corresponding controls. The significance for this assay was set at >2 fold changes, comparing between the control (untreated) group and the treated groups.

The effects of apoptotic related genes and proteins in both U-2 OS and MG-63 upon treatment with DK1 was subsequently assessed by qPCR and human apoptosis proteome profiler. After 48 h of treatment with DK1 (IC_50_), there was a substantial increase in the expression of caspase 3, caspase 9, caspase 8, BAX, Cdk 2, and cyclin A in U-2OS, as compared to MG-63 (Figure 6). Moreover, in Table 2 the levels of human pro-apoptosis protein expression in U-2OS like cleaved caspase 3, Bax, HTRA2/Omi, SMAC/Diablo, and cytochrome c were increased when treated with DK1 (IC_50_) whereas in MG-63 the levels of expression were decreased.

## 3. Discussion

Several plans have been undertaken to improve the biological activities of dietary curcumin. These include the synthesis of curcumin analogs and derivative that are mainly focused to enhance the clinical potential of dietary curcumin [8,10]. There are several reported studies that showed these chemically synthesized analogs, pose better selectivity towards several cancer cell lines like human colorectal (HCT-15), glioblastoma (U-251 MG), and human chronic myelogenous leukemia (K562) [8]. Thus, by using a chemical modification approach, lower molecular weight curcumin analog DK1 was synthesized [6]. This curcumin analog DK1 has been reported to possess anti-cancer activity by inducing apoptosis and cell cycle arrest towards breast cancer cell line like MCF-7 [6]. So, in this study the potential cytotoxic effect and the mechanism of apoptosis induction were further investigated in osteosarcoma cell lines in order to evaluate whether this curcumin analog DK1 is also selective towards other cancer types. Two osteosarcoma cell lines were used in these studies which are U-2 OS and MG-63 and the difference between the cell lines is the aggressiveness; U-2 OS is more aggressive due to its capability to invade and migrate at a higher rate compared to MG-63 [20].

As evidenced by the preliminary MTT assay, the curcumin analog DK1 successfully inhibited the proliferation of both U-2 OS and MG-63 in a dose-dependent manner. As illustrated in Table 1, the IC_50_ value showed that the curcumin analog DK1 poses a cytotoxic effect and inhibited the cell proliferation in U-2 OS better than in MG-63, without interfering with the proliferation of normal fibroblast 3T3. The MTT result also showed that DK1 has better cytotoxic effects towards osteosarcoma cell lines compared to natural curcumin. Based on the previous study conducted on human osteosarcoma cell lines, natural curcumin required 72 h incubation time to exhibit 50% inhibition concentration (IC_50_); U-2 OS (22.17 µM), MG-63 (22.77 µM) compared to DK1 which required only 48 h to exhibit the same cytotoxic effect. This result also corresponds to the study conducted on MCF-7 breast cancer cell line that showed DK1 has a better cytotoxic effect compared to natural curcumin [6,21]. To further confirm that DK1 induces apoptosis, morphological changes were observed using AO/PI double staining (Figure 2) where distinct feature of apoptosis appeared such as membrane blebbing, chromatin condensation, and cell shrinkage which confirmed apoptosis [22]. Based on Annexin V/FITC analysis also (Figure 3), curcumin analog DK1-treated cells showed a shifted pattern of phosphatidylserine externalization due to membrane disintegration which indicates DK1 induced apoptosis in both cell lines [19]. These results confirmed the perception that the mode of cell death is via apoptosis which conforms to previous study conducted on breast cancer cell line MCF-7 [6].

To further evaluate the mode of cell death, the effects of DK1 on the cell cycle activity were analyzed. During cell cycle progression there is a regulatory pathway called cell cycle checkpoints [17]. These checkpoints play a significant role in arresting the cells temporarily which allow the cells to repair the cellular damage like DNA mutation and activate the cell death program if the damage cannot be reversed [17]. Defects in these cell cycle checkpoints can be observed in many cancer types like osteosarcoma, which leads to irregular activity of the cell cycle that enhances the cancer proliferation [17,23]. Therefore, disruption of the cell cycle activity of cancer will lead to cell cycle arrest and increase the cell population in sub G0/G1 phase which demonstrates programmed cell death was activated via apoptosis [23,24]. Based on Figure 4, significantly higher cell cycle arrest at the S-phase can be observed in MG-63 compared to U-2 OS and this result was supported by down-regulated mRNA expression of Cdk 2 and cyclin A in MG-63 (Figure 6). Cdk 2 and cyclin A play a major role in S phase cell cycle progression and these two proteins will form a complex and phosphorylate certain targets that are involved in DNA replication [25]. Hence, this shows that DK1 is able to down-regulate the mRNA expression of these two genes, resulting in inhibition of cell cycle progression and DNA replication which lead to S phase arrest. This result corresponds to the previous report which indicated that, curcumin induced G1/S phase arrest in human osteosarcoma [26]. Even though there is no significant cell cycle arrest that can be observed in U-2 OS, it still has high accumulation of cell population at sub G0/G1 compared to MG-63. This result indicates that curcumin analog DK1 is more potent towards highly metastasis U-2 OS compared to MG-63, because when the DK1 concentration was increased in a dose dependent manner, it induced DNA fragmentation directly rather than cell cycle arrest.

There are two main pathway involved in apoptosis such as extrinsic pathway (death receptor) and intrinsic pathway (mitochondrial-dependent) [27]. As demonstrated by the JC-1 assay (Figure 5), DK1 showed that it is capable to trigger the depolarization of mitochondrial membrane potential in both U-2 OS and MG-63 which was proved by the decrease in the ratio of red/green. These results suggest that curcumin analog DK1 may induce cell apoptosis via the mitochondria-dependent pathway. Despite all this, to use JC-1 analysis as the main indicator to measure the mitochondrial membrane potential is not sufficient. Thus, expression of human related apoptosis proteins and genes were quantified in order to elucidate the mechanistic activity of curcumin analog DK1. Programmed cell death is a form of apoptosis that is mainly regulated by the caspase cascade and Bcl-2 family protein [28]. In the mitochondria-dependent or intrinsic pathway the membrane of mitochondrial is permeabilized when there is a receptor-independent stimulus like drugs and radiation. This causes the mitochondrial swelling which would eventually rupture the membrane resulting in apoptosome formation and activation of caspase 9 [27,28]. Activation of caspase 9 will lead to subsequent activation of effector caspase, like caspase 3 [28]. While for extrinsic pathway exposure to anti-cancer drugs will intervene with the cell surface of the death receptor which activates caspase 8 and subsequently activates the effector caspase [21]. As depicted in Figure 6, the mRNA expression of caspase 9, caspase 3, and Bax were up-regulated in U-2 OS, while for MG-63 the up-regulated mRNA expression involved caspase 9, caspase 3, and cytochrome c. These results suggest that both osteosarcoma cell lines undergo intrinsic apoptosis pathway upon treatment with curcumin analog DK1.

The anti-osteosarcoma activity of curcumin analog DK1 was further studied using human apoptosis proteome profiler. DK1 was able to enhance several pro-apoptotic proteins and inhibit anti-apoptotic protein. From Table 2, it can be seen that after treatment with DK1 several pro-apoptotic proteins were up-regulated like pro-caspase 3, cleaved caspase 3, Bax, cytochrome c, Fas, HTRA2/Omi, and SMAC/Diablo. Bax is member of the Bcl-2 protein family that is widely known as a pro-apoptotic protein. With the presence of apoptotic stimuli, Bax will regulate the mitochondrial potential which results in secretion of cytochrome c and another pro-apoptotic protein [29]. Upon activation of cytochrome c, this will stimulate the caspase activation like caspase 9 and 3 and lead to cell death [30,31]. Other pro-apoptotic proteins which showed up-regulated expressions like HTRA2/Omi and SMAC/Diablo also played a significant role in the induction of cell death via apoptosis. Both of these proteins were mitochondrial protein that bind to the inhibitor of apoptosis proteins (IAPs) and release caspase proteins to activate apoptosis [7,32,33]. Furthermore, DK1 also inhibited the anti-apoptosis proteins like HO-1/HMOX1/HSP32 which provide a cytoprotective effect for cancer cells against apoptosis [34]. However, only in U-2 OS the up-regulated expression of pro-apoptotic protein was manifested compared to MG-63 where most of the protein did not show any sign of change in expression except for cleaved caspase 3.

## 4. Materials and Methods

### 4.1. Preparation of Curcumin Analogue DK1

Curcumin analog DK1 was obtained from Dr. Muhammad Nadeem Akhtar from Universiti Malaysia Pahang, who synthesized the DK1 by using the outlined protocol by Ali et al. [6].

### 4.2. Cell Culture

U-2OS cells were maintained in McCoy’s 5A culture media, while MG-63 cells were maintained in Dulbecco’s Modified Eagle's Medium (DMEM). Both were supplemented with 10% fetal bovine serum and 1% of penicillin/streptomycin in 25 cm^2^ flask at 37 °C, 5% CO_2_ environment. After reaching 80% confluence, TryplE was used to harvest the cells for analysis.

### 4.3. Cell Viability Assay

Through the reduction of the 3-(4,5-dimethylthiazol-2-yl)-2,5-diphenyltetrazolium bromide (MTT), insoluble formazan could be measured in live cells and was used to determine cell viability [35]. Briefly, U-2OS cells at a concentration of 4 × 10^4^ cell/mL and 8 × 10^4^ cell/mL MG-63 were seeded in a 96-well plate and were both allowed to attach overnight. Then, the cells were treated with difference concentrations of DK1 (0–30 µg/mL) and incubated for 24 h, 48 h and 72 h. Doxorubicin was used as positive control. 20 µL of MTT (5 mg/mL) solution was added 4 h before the end of incubation times and dimethyl sulfoxide (DMSO) was used to solubilize the tetrazolium salt. By using the ELISA (Bio-Tek Instrument, Winooski, VT, USA), the wavelength of optical density was measured at 570 nm. The percentage of cell viability was determined by using the following formula:

Percentage of cell viability = (OD sample)/OD control × 100%

Concentration of the treatment that resulted in 50% inhibition of cell growth (IC_50_) was obtained when plotting the dose-response curve and was used as a cytotoxicity parameter. The IC_50_ values and concentration with the highest cytotoxic effect were used throughout the study to induce cell death.

### 4.4. Cell Treatment

Based on MTT assay results, three doses of DK1 were used for the remaining assay. The three doses were used to administer to U-2OS; IC_25_ (2.2 µM), IC_50_ (19.6 µM), IC_75_ (30 µM) respectively and to MG-63; IC_25_ (6.6 µM), IC_50_ (23.8 µM), IC_75_ (30 µM) respectively. DK1 was dissolved in dimethyl sulfoxide (DMSO) with the volume below 0.1%, since it was not soluble in water.

### 4.5. Florescence Detection Using Acridine Orange/Propidium Iodide (AO/PI) Double Staining Assay

Acridine orange/propidium iodide (AO/PI) double staining assay was performed on bone cancer cell line to determine the mode of cell death microscopically. Bone cancer cell lines were seeded in a 6-well plate at adensity of 6 × 10^4^ cells/well. For the qualitative assessment of apoptosis, cells were exposed to three different concentrations of DK1 and incubated for 48 h. Treatment-free culture was used as negative control and cells were detached and collected at the end of each incubation time. Then, cells were washed with PBS and incubated with a 1:1 ratio of acridine orange (10 µg/mL) and propidium iodide (10 µg/mL). An amountof 10 µL of incubated suspension cells were placed on a slide and viewed immediately under a fluorescent microscope (Nikon FC-35DX, Nikon, Tokyo, Japan) at 200× magnification with filter range 450–490 nm. The cells emitting green color with intact membrane and nuclei were counted as viable cells. Cells which emitted green color with distinct features such as membrane disruption and chromatin condensation were counted as apoptotic. Meanwhile, red-fluorescent cells with loss of membrane integrity were assigned as necrotic.

### 4.6. Cell Cycle Analysis

Bone cancer cell lines were seeded in a 6-well plate with density of 6 × 10^4^ cells/well. To elucidate cell cycle progression, flow cytometry cell cycle analysis was carried out by using a BD Cycletest™ Plus DNA Kit (BD Biosciences, San Jose, CA, USA). Treatment-free culture was used as negative control. After 48 h of treatment with three different concentrations of DK1, cells were collected, permeabilized, and fixed with buffer provided by the kit and incubated at −20 °C (minimum of 24 h). After 24 h, the fixed cells were pelleted and resuspended using 250 µL of solution A and incubated at room temperature for 10 min. Then the cells were further resuspended with 200 µL of solution B and incubated at room temperature for 10 min. Next the cells were stained using solution C that contained propidium iodide and incubated for 10 min. A NovoCyte^®^ Flow Cytometer (ACEA Biosciences, Inc., San Diego, CA, USA) was used to analyze the cell cycle activity. Minimum of 10,000 cells in the population were captured and the experiment was repeated three times with similar parameters.

### 4.7. Annexin V/FITC Binding Assay

Annexin-V FITC analysis was employed to verify the mode of cell death induced by DK1, by using a FITC Annexin-V Apoptosis detection kit (BD Biosciences). Similarly, the cells with density of 6 × 10^4^ cells/well were treated with three concentrations of DK 1. Treatment-free culture was used as negative control. Briefly, after the 48-h incubation time, cells were collected, pelleted and diluted up to a final concentration of 1 × 10^4^ cells/mL in 1XAnnexin-V binding buffer. Cells were aliquots and stained with 5 µL of PI and FITC Annexin-V for 15 min in the dark. Then, the cells were further diluted with 400 µL of 1XAnnexin-V binding buffer for the analysis using a BD Accuri™ C6 flow cytometer (Becton Dickinson, Franklin Lakes, NJ, USA). Approximately, 10,000 cells in the population were captured. The experiment was repeated three times with similar parameters.

### 4.8. JC-1 MitoScreen Assay

The depolarization of the mitochondrial membrane potential was measured by using the BD MitoScreen Kit (BD Biosciences). Bone cancer cell lines were seeded in a 6-well plate with a density of 6 × 10^4^ cells/well. The next day, the cells were exposed to three different concentrations of DK1. After 48 h, the cells were collected and centrifuged at 2000× *g* for 5 min. Around 1 × 10^6^ cells were incubated with 500 μL of JC-1 working solution. The JC-1 working solution was prepared in accordance of 1:100 ratios of JC-1 stock solution and assay buffer. This working solution was incubated at 37 °C for 15 min. Then, the cells were washed twice using the assay buffer, before proceeding to the BD Accuri™ C6 analysis (Becton Dickinson).

### 4.9. Quantitive Real Time PCR Assay

QIAGEN RNeasy Kit (Qiagen, Hilden, NRW, Germany) was used to isolate the total RNA by following the manufacturer’s protocol. By using spectrophotometer (Beckman Coulter, Brea, CA, USA), the concentration and purity of isolated RNA were measured and 1% of agarose gel was run to determine the integrity of isolated RNA. Then, 5 µg of isolated RNA was converted to cDNA using RevertAid First Strand cDNA Synthesis Kit (Thermo Scientific, Waltham, MA, USA) by following the manufacturer’s protocol. The accession number and primer sequence used in gene expression analysis are given in Table 3. Next, real-time PCR was carried out using Thermo Scientific Luminaris Color Hi Greenq PCR Master Mix (Thermo Scientific, Waltham, MA, USA) on the Eco™ Real-Time PCR System (Illumina, San Diego, CA, USA).The PCR reaction program was initiated at 95 °C for 10 min, followed by denaturation at 95 °C for 10 s and annealing/extension at 58 °C for 30 s. These denaturation and extension phases were repeated for 40 cycles. The qPCR result was analyzed using EcoStudy Software v4.0 (San Diego, CA, USA) based on the primers efficiency and normalized to two housekeeping genes; ACTB and 18srRNA. Based on the normalized result, the difference in the fold change value was calculated by comparing between the untreated/control group and the DK1-treated group.

### 4.10. Proteome Profiling of Apoptosis-Related Protein

The expression of apoptosis-related protein was evaluated on the bone cancer cell lines treated with DK1 (IC_50_). Proteome profiler was performed using Human Apoptosis Array Kit (R&D Systems, Milpitas, CA, USA) and this array kit was able to detect 35 human apoptosis-related proteins simultaneously. Protein from bone cancer cell lines was extracted using 600 µL of RIPA buffer (50 mM Tris-HCl, 150 mM NaCl, 1.0% TritonX-100, 0.5% sodium deoxycholate, 0.1% SDS) and supplemented with 10 mg of pre-made protease inhibitor cocktails (Roche, Basel, Switzerland). Then the protein that had been extracted could be quantified using Bradford assay (Sigma, St. Louis, MO, USA). The membrane was incubated for 1 h and soaked in 2 mL of array buffer placed on a shaker that served as a blocking buffer. One mL of Lysis Buffer 17 was added to the protein lysate to prepare the protein sample. The prepared sample was added into the membrane and incubated at 4 °C overnight. The membrane was then washed three times with 20 mL of wash buffer provided with the kit. Later, the membrane was transferred into the 4-well multi dish that contained reconstituted detection antibody cocktails and incubated on the shaker for 1 h. The membrane was washed three times again using the wash buffer. Diluted 2 mL Streptavidin-HRP was poured with array buffer on the membrane and incubated for 30 min. Finally, the membrane was washed three times before 1 mL of Chemi reagent Mix was pipetted onto the membrane for viewing. The membrane was scanned using the ChemiDoc XRS (BioRad, Hercules, CA, USA).

### 4.11. Statistical Analysis

The data was presented as statistical means ± standard error mean (S.E.M) from three independent experiments. SPSS version 20 (SPSS Inc., Chicago, IL, USA) was used to perform all statistical analysis. The statistical comparison analysis was done using the one-way ANOVA, followed by Tukey’s post hoc test. Statistically significant data was considered when *p* < 0.05.

## 5. Conclusions

Based on this study it can be concluded that curcumin analog DK1 was found to be more sensitive to highly metastasis U-2 OS cell line by induction of apoptosis. This observation was proved by up-regulated expression of pro-apoptotic genes and protein like caspase 9, caspase 3, Bax, and cytochrome-c in U-2 OS cells. Further study needs to be conducted to elucidate the in vivo efficacy which could strengthen the potential of DK1 as an anti-osteosarcoma drug.

## Figures and Tables

**Table 1:** The IC_50_ value of DK 1 on osteosarcoma cell lines (MG-63 and U-2OS) and normal fibroblast cell line to be used in other assays.

Cell Lines	IC_50_ (µM)
MG-63	23.8 ± 0.8
U-2OS	19.6 ± 0.3
3T3	>30

Notes: Cytotoxicity effects of DK1 on osteosarcoma cell lines and normal fibroblast cell line. The IC_50_ values of DK1 in MG-63 and U-2OS at 48 h post-treatment. The IC_50_ value for 3T3 after treated with DK1 for 48 h. All data were expressed as mean ± standard error mean (S.E.M).

**Table 2:** Apoptosis pathway-related protein expression in DK1 treated U-2 OS and MG-63.

Cell Lines	Proteins	Relative Intensity (Fold Change)	Regulation
U-2 OS	Pro-caspase 3	2.0 * ± 0.15	Up
	Cleaved caspase 3	4.6 * ± 0.07	Up
	Bax	4.5 * ±0.38	Up
	Cytochrome c	3.0 * ± 0.35	Up
	Fas	2.2 * ± 0.05	Up
	HO-1/HMOX1/HSP32	−0.6 ± 0.05	Down
	HTRA2/Omi	3.3 * ± 0.13	Up
	SMAC/Diablo	2.9 * ± 0.04	Up
MG-63	Pro-caspase 3	−0.6 * ± 0.03	Down
	Cleaved caspase 3	1.4 * ± 0.12	Up
	Bax	−0.8 ± 0.02	Down
	Cytochrome c	−0.8 ± 0.08	Down
	Fas	−0.4 * ± 0.01	Down
	HO-1/HMOX1/HSP32	−0.4 * ± 0.13	Down
	HTRA2/Omi	−0.6 * ± 0.12	Down
	SMAC/Diablo	−0.8 *± 0.01	Down

Note: Human apoptosis proteome profiler of the OS cell lines treated with DK1 (IC_50_) for 48 h. All data are expressed as mean ± standard error mean (S.E.M). (−) the symbol indicates the expression was down-regulated. * *p* < 0.05 compare with corresponding controls.

**Table 3:** The accession number and primer sequence used in gene expression analysis.

Genes	Accession Number	Forward Primers	Reverse Primers	Amplicon Size (bp)
*Caspase 3*	NM_004346	AGAACTGGACTGTGGCATTGAG	GCTTGTCGGCATACTGTTTCAG	191
*Caspase 9*	NM_001229	TGTCCTACTCTACTTTCCCAGGTTTT	GTGAGCCCACTGCTCAAAGAT	101
*Caspase 8*	NM_001228	CATCCAGTCACTTTGCCAGA	GCATCTGTTTCCCCATGTTT	128
*BAX*	BC014175	CAAGAAGCTGAGCGAGTGT	CAGTTGAAGTTGCCGTCAGA	153
*Cytochrome C*	NM_018947.5	GGGCCAAATCTCCATGGTCT	GGCAGTGGCCAATTATTACTC	246
*Cyclin A*	NM_001237 212	GATGCTGACCCATACCTCAAG	GGTGAAGGTCCATGAGACAAG	160
*CDK 2*	NM_052827	GAAGATGGACGGAGCTTGTT	TGGAGGAGAGGGTGAGATTAG	173
*ACTB*	NM_001101.3	AGAGCTACGAGCTGCCTGAC	AGCACTGTGTTGGCGTACAG	184
*18srRNA*	X03205	GTAACCCGTTGAACCCCATT	CCATCCAATCGGTAGTAGCG	151
